# Treatment of Stainless Steel Rinse Waters Using Non-Dispersive Extraction and Strip Dispersion Membrane Technology

**DOI:** 10.3390/membranes13120902

**Published:** 2023-12-06

**Authors:** Francisco Jose Alguacil, Jose Ignacio Robla

**Affiliations:** Centro Nacional de Investigaciones Metalurgicas (CSIC), Avda. Gregorio del Amo 8, 28040 Madrid, Spain

**Keywords:** rinse water, DP8R, non-dispersive extraction and strip dispersion, separation, liquid membrane

## Abstract

The extraction of Fe(III), Cr(III), and Ni(II) from stainless steel rinse water using non-dispersive extraction and strip dispersion membrane technology was carried out in a microporous hydrophobic hollow-fibre module contactor. The fibres were of polypropylene, whereas the organic extractant DP8R (bis(2-ethylhexyl) phosphoric acid) diluted in ExxsolD100 was used as the carrier phase. The rinse water containing the three elements was passed through the tube side, and the pseudo-emulsion formed by the organic phase of DP8R in Exxol D100 and an acidic strip solution were passed through the shell side in a counter-current operation; thus, a unique hollow fibre module was used for extraction and stripping. In non-dispersive extraction and strip dispersion technology, the stripping solution was dispersed into the organic membrane solution in a vessel with an adequate mixing device (impeller) designed to form strip dispersion. This pseudo-emulsion was circulated from the vessel to the membrane module to provide a constant supply of the organic phase to the membrane pores. Different hydrodynamic and chemical variables, such as variation in feed and pseudo-emulsion flow rates, strip phase composition, feed phase pH, and extractant concentration in the organic phase, were investigated. Mass transfer coefficients were estimated from the experimental data. It was possible to separate and concentrate the metals present in the rinse water using the non-dispersive extraction and strip dispersion technique.

## 1. Introduction

When an aqueous solution to be treated has a low metal concentration, conventional liquid–liquid extraction may not be the most suitable technology for this operation. This unsuitability is mostly due to losses of the organic phase to the raffinate; thus, options to take advantage of the selectivity presented by the organic extractants were developed. Among them, membrane extraction using microporous materials to immobilize the aqueous/organic interface within the membrane pores was developed. In this operation, the solute (metal) is transported from the aqueous solution to the organic phase through the membrane without phases of dispersion. Moreover, this type of technology combines the extraction and stripping operations in one single step.

A further development in these membranes technologies was the non-dispersive extraction and strip dispersion (also known as pseudo-emulsion-based strip dispersion, etc.), which provided the system with a good level of stability and performance. This technique varies from the conventional membrane operation (as described above) in which the strip solution is dispersed into the organic phase, and this pseudo-emulsion is in contact with the membrane pores; due to the hydrophobicity of such pores, the organic phase is wetted, and it is supported into the membrane micropores, making possible the transport of the solute from the aqueous feed solution to the organic phase, and from this to the stripping phase. This stripping operation is favoured by the continuous mixing of both organic and stripping phases. Once the operation is finished, the mixing is stopped, the organic and strip phases rapidly disengage, the feed phase is depleted of solute, and the stripping phase concentrates on the solute.

Using one or another type of liquid membrane operations, there were recent references to its applicability for the recovery and/or elimination of metals from different aqueous media [1,2,3,4]. Besides the above, most recent references to supported liquid membrane operation using permeation cells investigations have included the transport of Cr(VI) using organic solutions of the commercially available Cyanex 921 and Cyanex 923 phosphine oxides [5]; also, the transport of In(III) from HCl solutions has been investigated using the same methodology and the pseudo-protic ionic liquids TOAH^+^Cl^−^ and TODAH^+^Cl^−^ as carriers [6]. The ionic liquids [omim^+^][PF_6_^−^] (1-octyl-3-methylimidazolium hexafluorophosphate) and methyl trioctylammonium chloride [MTOA^+^][Cl^−^] have been investigated in the separation of Zn(II), Fe(III), Cd(II), and Cu(II) from HCl solutions [7]; in this case, the ionic liquids were from part of the membrane (polymer inclusion membranes) instead of being supported within the pores of the membrane support. Another liquid membrane technology, such as the electromembrane extraction technique, was used in the removal of Cu(II) from simulated wastewaters [8]; this investigation included the use of two carriers (tris(2-ethylhexyl) phosphate (TEHP) and bis(2-ethylhexyl) phosphate (DEHP)), and voltage was applied between the graphite anode (inserted in the donor phase semi-cell) and the stainless steel cathode (inserted in the receiving or stripping phase half-cell). A supported liquid membrane containing DEHPA (the acidic form of DEHP) in kerosene as the carrier solution was used in the transport of Nd(III) and Er(III) under different experimental conditions [9].

Using hollow fibre modules, the transport of Fe(III) using a pseudo-protic ionic liquid derived from the primary amine Primene JMT and sulphuric acid was investigated [10]; in this work, acidic strip dispersion was used to recover the metal from loaded organic phases. The same hollow fibre strip dispersion technology was used to extract Pd(II) and Pt(IV) from synthetic and real solutions using alkoxymine-1-propylpyridinium derivatives [11]. Hollow fibre modules in supported liquid membrane configuration were recently used in the non-dispersive extraction of Cu(II) and Zn(II) using trifluoroacetylacetone as the carrier [12]. Also, a quaternary ammonium salt (Aliquat 336) dissolved in toluene as the carrier phase immobilized in the pores of a hollow fibre module was used in the co-extraction of As(V) and Hg(II) [13]. A hollow-fibre-supported liquid membrane operation using sunflower oil as the carrier was used in the separation of Hg(II) from simulated wastewaters [14]. Using the same supported liquid membrane operation as in the above reference, the transport of Sm(III) was investigated through the use of DEHPA (bis(2-ethylhexyl) phosphoric acid) and EHEHPA (mono 2-ethylhexyl ester) [15].

In the production of stainless steel and after the pickling step, the steel surface must be rinsed with water in order to clean it and to get rid of all acids on the steel surface. From this rinse operation, the exhausted water contains iron(III), chromium(III), nickel(II), and nitric and hydrofluoric acids in different compositions. Although neutralization of these waters with lime commonly occurs prior to its discharge, more stringent legislation regarding the discharge of nitrates and other pollutants has led to investigations into the use of more advanced and environmentally friendly techniques for the treatment of these rinse waters. One of the proposed methods combined the use of a membrane (filtering, reverse osmosis, electrodyalisis) and ion exchange technologies [16,17] though the use of liquid–liquid extraction [18], and other technologies [19] have been proposed.

This manuscript investigates the use of an advanced liquid membrane technology, non-dispersive extraction and strip dispersion, using a hollow fibre module to treat rinse water for an industrial plant. Different variables were investigated, including hydrodynamic and chemical conditions, flow rates, pH, compositions of the feed phase, extractant concentration in the organic phase, etc. Under the most adequate experimental conditions, it was possible to separate and/or concentrate the various metals present in the rinse water.

## 2. Materials and Methods

### 2.1. Materials

The commercial acidic extractant DP8R (bis(2-ethylhexyl) phosphoric acid) was obtained from Daihachi (Japan), with a molecular weight of 322 and a density of 0.98 g/cm^3^ (20 °C). It was used without further purification. Exxsol D100 (99% aliphatics) obtained from Exxon Chem Iberia, Spain, was used to dissolve the extractant.

The original rinse water proceeded from a Swedish plant, and it was subjected to a treatment in order to recover both nitric and hydrofluoric acids [16], resulting in a solution containing 0.1 g/L each of Fe(III), Cr(III), and Ni(II). All other chemicals used in this work were of AR grade.

The hollow fibre module device used in the experimental work was obtained from Hoechst Celanese: Liqui-Cel 8 × 28 cm 5PCG-259 contactor and 5PC5-1002 Liqui-Cel laboratory LLE. The corresponding specifications were given in a previous publication [10].

### 2.2. Methods

**Figure 1:**
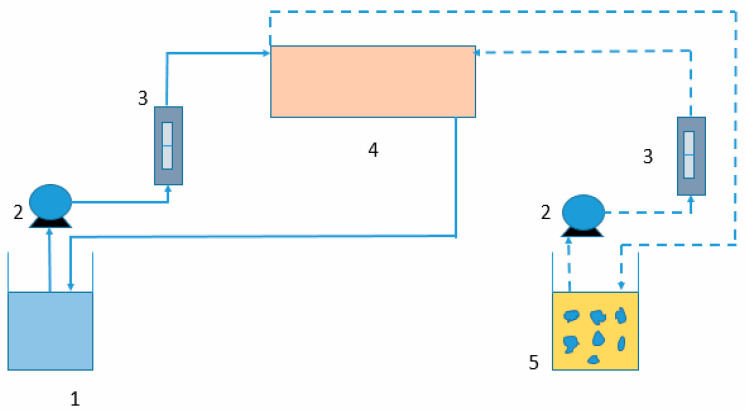
Schematic view of the hollow fibre membrane process for extracting metals contained in the rinse water. 1—feed phase vessel. 2—pumps. 3—flow-meters. 4—hollow fibre module. 5—pseudo-emulsion vessel.

The operational method was similar to that described in the literature [10]. A schematic view of the membrane operation, using one contactor in the recirculation mode of both the feed and pseudo-emulsion phases, is shown in Figure 1.

**Figure 2:**
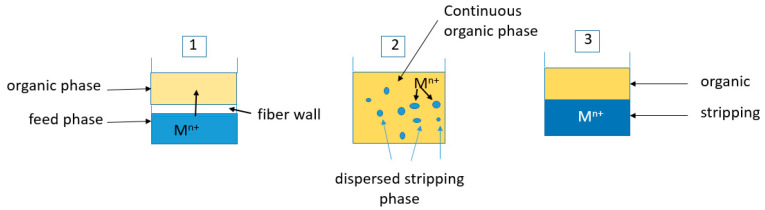
Details of the transfer process. 1—non-dispersive extraction in the hollow fibre module. 2—metal transfer in the pseudo-emulsion vessel. 3—distribution of phases in the pseudo-emulsion vessel after phases of disengagement.

Figure 2 shows a detailed view of the stripping process. The metal-loaded organic phase was put into intimate contact with the strip phase due to the continuous mixing of both phases, and the metals were being transferred from the organic to the strip phase (1). Once the operation finished, mixing was stopped, and both phases quickly disengaged (2), resulting in an organic phase depleted of metals, which can be used in a new extraction operation, and a stripping phase containing the metal(s) in a concentrated form.

In the operation, the volume of the feed phase was 4 L, whereas the volume of the pseudo-emulsion phase was 0.8 L (0.4 L each of the organic and stripping solutions). At elapsed times, aliquots of the feed and pseudo-emulsion vessels were taken and analysed for metal concentrations in the aqueous solutions through atomic absorption spectrometry (Perkin Elmer 1100B spectrophotometer, Oxford, UK). The percentage of metals extracted was calculated as:

(1) $$\%M=\frac{{\left[M\right]}_{f,0}-{\left[M\right]}_{f,t}}{{\left[M\right]}_{f,0}}\cdot 100$$

where [M]_f,t_ and [M]_f,0_ are the metal concentrations in the feed phase at an elapsed time and time zero, respectively. With respect to the reproducibility of data, several experiments were performed to check the feasibility and consistency of results under the same experimental conditions. Reproducibility was found to be good enough for the results obtained for a fixed period of time using three sets of data. The overall permeation coefficient (P) was calculated using the next equation:

(2) $$\ln\frac{{\left[M\right]}_{f,t}}{{\left[M\right]}_{f,0}}=-\frac{A\cdot P}{V}t$$

where A was the membrane area (1.4 m^2^), V was the volume (4 L) of the feed phase, and t was the elapsed time. Based on three observations, the *p* value exhibited a variation of ±2%.

## 3. Results and Discussion

The extraction of metals using an organic derivative of phosphoric acid, such as DP8R, responded to a cation exchange mechanism that, in a general form, can be described by the next equilibrium [20]:

(3) $$M_{aq}^{n+}+nHR_{\mathrm{org}}\Leftrightarrow MR_{n_{\mathrm{org}}}+nH_{aq}^{+}$$

where HR represents the active group of the extractant, and the subscripts aq and org represent the respective aqueous and organic phases. Thus, extraction proceeded at high pH values (low acidic concentrations), shifting the equilibrium to the left, and stripping was carried out using more concentrated acid solutions, shifting the equilibrium to the right.

However, and due to the possibility of the extractant dimerization [21], the above equilibrium takes a most elaborated form in function of the oxidation state of the metal [22]:

(4) $$M_{aq}^{2+}+n{\left(HR\right)}_{2_{\mathrm{org}}}\Leftrightarrow MH_{2\left(n-1\right)}R_{2n_{\mathrm{org}}}+2H_{aq}^{+}$$

(5) $$M_{aq}^{3+}+n{\left(HR\right)}_{2_{\mathrm{org}}}\Leftrightarrow MH_{2n-3}R_{2n_{\mathrm{org}}}+3H_{aq}^{+}$$

where n is a stoichiometric factor that depends on the metals extracted in the organic phase.

### 3.1. Evaluation of DP8R as an Extractant (Carrier) for Fe(III), Cr(III), and Ni(II)

**Figure 3:**
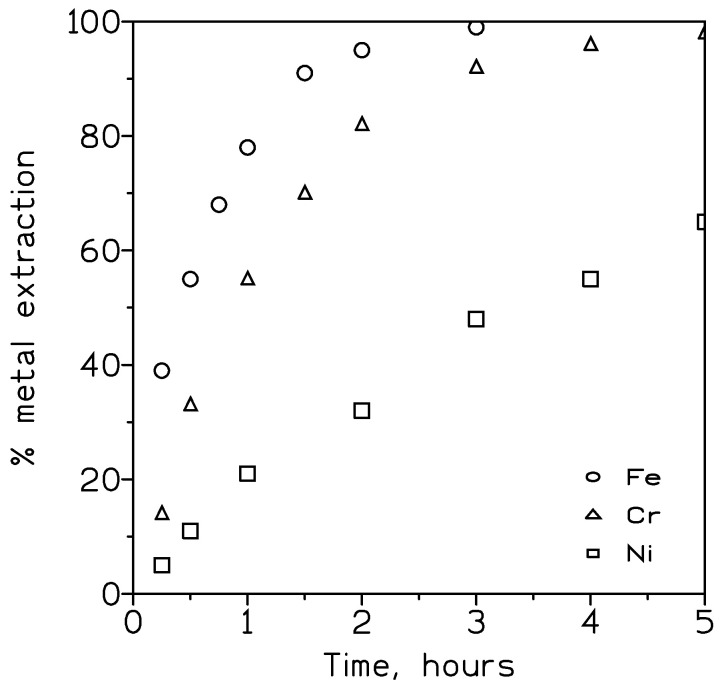
Extraction of single metals using DP8R. Feed flow: 34 cm^3^/min. Pseudo-emulsion flow: 180 cm^3^/min. Temperature: 20 °C.

A series of tests were conducted to investigate the performance of the extractant in the removal of these metals from the solution. Firstly, experiments were carried out on solutions that contained single metals; thus, the feed solution contained 0.1 g/L of each metal (separately) at pH 3.0, whereas the pseudo-emulsion phase contained 20% *v*/*v* DP8R in Exxsol D100 and 1 M of sulphuric acid as the strippant. The results of these experiments are shown in Figure 3.

It can be seen that from the beginning of the experiment, iron(III) was extracted preferably to chromium(III) and nickel(II), reaching 99% extraction after three hours against 90% chromium(III) extraction at this time. Nickel(II) was extracted at a rate of 65% after five hours of reaction time. Within the extraction data, the overall permeation coefficient for each metal was estimated using Equation (2). Table 1 summarizes these values together with the rate of metal recovery in the strip solution.

These results showed that both chromium(III) and nickel(II) can be stripped from the organic solution using sulphuric acid, but not iron(III). Also, and as expected from the results shown in Figure 1, the permeation order followed the series Fe(III) > Cr(III) > Ni(II).

**Figure 4:**
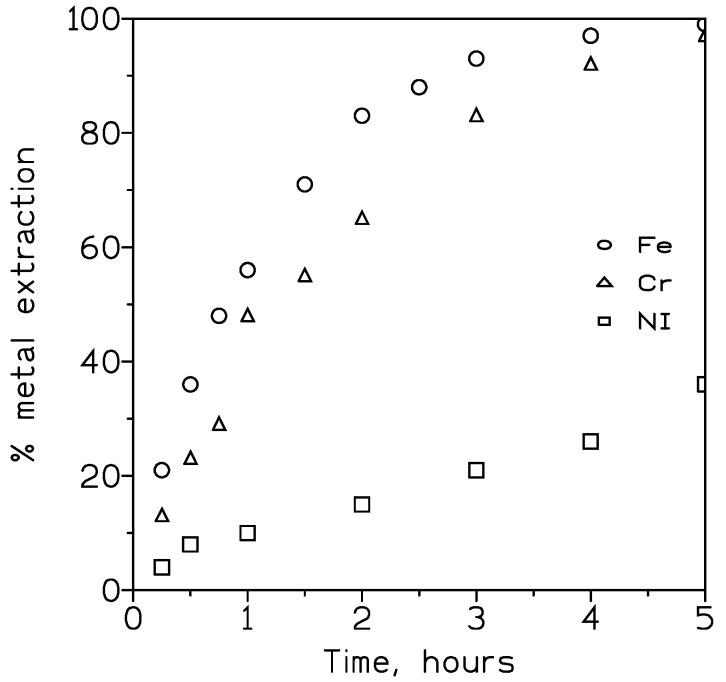
Metal extraction using DP8R in ExxsolD100 from a multi-elemental solution. Experimental conditions as in Figure 1.

Also, the performance of the extractant was investigated when the feed phase (rinse water) contained the three metals together. The experimental conditions were the same as those used in Figure 1, and the percentage of metal extraction against time is plotted in Figure 4.

These results indicated that in this multi-elemental solution, the rate of iron(III) extraction was greater than that of chromium(III) after four hours of reaction time. In any case, both metals were nearly quantitatively extracted (99%) from the feed phase after 5 h. In the case of nickel(II), 36% of metal extraction was reached after the fifth hour of reaction. The respective overall permeation coefficients and the rate of recovery in the strip solution are given in Table 2.

Again, it was shown that both chromium(III) and nickel(II) can be recovered from the organic phase using sulphuric acid as the strippant; in the case of iron(III), and as shown in Table 1, this acidic chemical was ineffective in recovering the metals from the loaded organic phase.

Comparison of the overall permeation coefficient values in Table 1 and Table 2 shows that in the case of the multi-elemental solution (Table 2), these values were lower than the respective ones obtained when single-metal solutions were used in the experiment (Table 1). This decrease was attributable to the population or crowding effect [23] produced by the presence of various solutes in the feed phase. This crowding effect was not predictable, and when occurs, it must be experimentally determined for each solute-carrier phase system.

### 3.2. Effect of Varying the Feed Phase Flow Rate on Metal Extraction

Once it was established that DP8R can be used to extract (transport) the various metals present in the rinse water, further investigation was performed to study the influence of several variables on metal removal from the feed phase.

Firstly, the influence of the feed phase flow rate on Fe(III), Cr(III), and Ni(II) extraction (transport) across the fibres impregnated with a solution of DP8R in Exxsol D100 was investigated. The feed phase contained 0.1 g/L each of the three elements at pH 2.5, whereas the pseudo-emulsion phase was composed of an organic phase of 20% *v*/*v* DP8R in the organic diluent and a stripping solution of 1 M of sulphuric acid. Feed phase flow rates varied between 17 and 120 cm^3^/min, and the pseudo-emulsion flow rate was maintained constant at 180 cm^3^/min.

**Figure 5:**
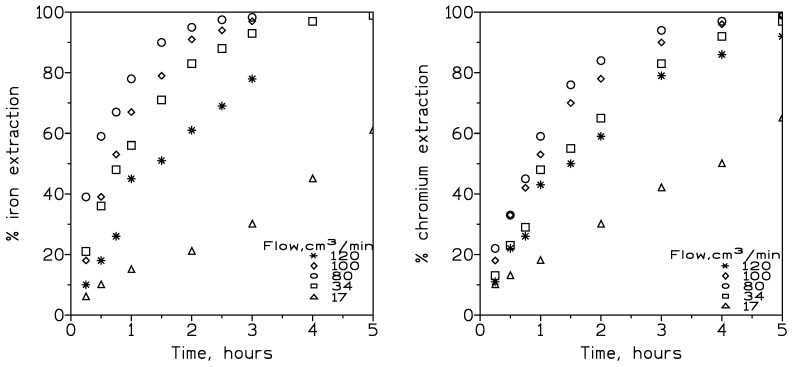
Iron(III) (**left**) and chromium(III) (**right**) extraction at various flow rates. Temperature: 20 °C.

Figure 5 and Table 3 show the results of this investigation.

Figure 5 shows that both iron(III) and chromium(IIII) increased their respective percentages of extraction at a fixed time, with the increase in the feed flow rate up to 80 cm^3^/min, and then it decreased. At 80 cm^3^/min, 95% extraction can be reached after 2 and 3 h for iron(III) and chromium(III), respectively. This behaviour was reflected in the increase in the overall permeation coefficient values, from 17 to 80 cm^3^/min, and, from then, in a decrease of these values at higher flow rates. It was described [24] that two types of diffusional resistances can be found in the transport process of solutes across an organic phase supported in the pores of a membrane. These resistances were due to (i) the feed phase boundary layer and (ii) the membrane.

Thus, at 80 cm^3^/min, the thickness of the feed phase boundary layer was minimized, and, as a consequence, the feed phase resistance to metal transport was also minimized; this resulted in consideration of the fact that the diffusion contribution of metal species present in the feed phase to the transport phenomena was constant [25]. However, the appearance of this minimum thickness of the boundary film did not imply the complete elimination of the aqueous diffusion layer, although, as said above, its resistance was minimized [26].

The decrease in metal transport at flow rates exceeding 80 cm^3^/min can be attributable to various effects: (i) the increase in the turbulence created in the feed phase at these higher flow rates, which resulted in a displacement of the organic phase trapped in the membrane pores (this removal contributed to making difficult the maintenance of the interface within the pore limits), and (ii) the direct relationship between the increase in the feed flow and a lower residence time of the feed solution in the hollow fibre module, and a third effect due to the possible formation of stable emulsions along the tube side of the fibres as the feed flow increased [27].

In the case of nickel(II) extraction, this metal was extracted at a maximum rate of 22% and five hours of reaction time. Thus, it was evident that nickel was transported at a much lower rate than iron(III) and chromium(III), i.e., at 80 cm^3^/min, and the overall permeation coefficient values for nickel(II), iron(III), and chromium(III) were 5.1 × 10^−6^, 1.1 × 10^−4^, and 7.1 × 10^−5^ cm/s, respectively. In terms of metal separation, and considering the separation factor (SP) as the ratio of the permeation coefficients:

(6) $$SP=\frac{P_{M1}}{P_{M2}}$$

the values of the SP for the systems Fe-Ni and Cr-Ni were 22 and 14, respectively, indicating the possibilities for the separation of iron(III) and chromium(III) from nickel(II) using the present experimental conditions.

The metal concentration factor was evaluated and defined as the ratio of the metal concentration in the stripping phase at the end of the experiment to the initial metal concentration in the feed phase. The chromium(III) concentration in the strip solution was around nine times of the initial metal concentration in the feed phase. The feasibility of recovering metals from the rinse water with this membrane operational mode using DP8R in Exssol D100 as the carrier phase was proved. It should be noted here that as iron(III) was not stripped with sulphuric acid, this concentration factor was not considered here (see below for iron(III) stripping).

In order to check the reproducibility of the set-up, a number of metal permeability values were evaluated using the same experimental conditions as above. The values for the three different sets were found to be 0.99 × 10^−4^ cm/s, 1.12 × 10^−4^, and 1.13 × 10^−4^ cm/s for iron(III) and 6.99 × 10^−5^, 7.11 × 10^−5^, and 7.24 × 10^−5^ cm/s in the case of chromium(III).

### 3.3. Influence of the Pseudo-Emulsion Phase Flow Rate and Composition on Metal Transport

The influence of varying the pseudo-emulsion flow rate on the transport of the metals presented in the feed phase was investigated. For these experiments, the feed and pseudo-emulsion phases were similar to those used in Section 3.2, whereas the feed phase flow rate was maintained constant at 80 cm^3^/min and the pseudo-emulsion phase flow rate was varied in the 100–180 cm^3^/min range. The results from this investigation showed that the variation of the pseudo-emulsion flow rate had a negligible effect on the extraction (transport) of the metals.

Using the same experimental conditions as in Section 3.2, the variation of the composition of the stripping phase of the pseudo-emulsion was investigated. The use of sulphuric acid solutions in the 0.5–2 M concentration range did not affect the metal extraction, and it also had a negligible effect on the percentages of metal recoveries in the stripping phase (around 3% for iron(III) and around 90% for chromium(III) and nickel(II)). As it was mentioned above, sulphuric acid was not effective in the recovery of iron(III) from the loaded organic phase; the recovery of this element from these organic phases is described in a further subsection.

### 3.4. Influence of the pH Value of the Feed Phase on Metal Transport

In these cationic exchange systems, the proton concentration gradient between the feed and the stripping phases must be one of the driving forces to consider for the permeation of the metal ion.

Pseudo-emulsion phases composed of 20% *v*/*v* DP8R in Exxsol D100 and 1 M of sulphuric acid and feed phases of 0.1 g/L each of Fe(III), Cr(III), and Ni(II) at various pH values (1–3) were used to investigate the effect of this variable in the transport of the three metals.

**Figure 6:**
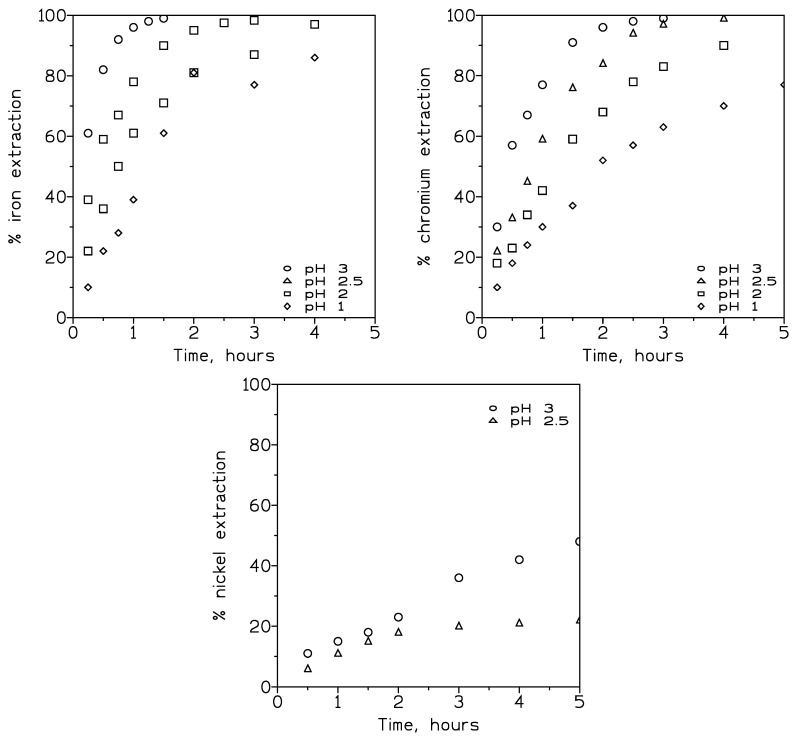
Metal extraction at various pH values of the feed phase. Iron(III) (**upper left**). Chromium(III) (**upper right**). Nickel(II) (**bottom centre**). Feed flow: 80 cm^3^/min. Pseudo-emulsion flow: 180 cm^3^/min. Temperature: 20 °C.

Figure 6 shows the variation in the percentage of extraction of iron(III) (left) and chromium(III) (right) with time, whereas Table 4 summarizes the values of the overall permeation coefficients and the recoveries of the elements in the stripping solution at the end of the experiments.

From Figure 6, it can be seen that the pH value of the feed phase greatly influenced the extraction, and, consequently, the transport of both iron(III) and chromium(III), thus increasing this extraction as the pH of the solution increased. Obviously, this was due to the fact that, in accordance with Equations (3)–(5), a decrease in the proton concentration of the aqueous phase produced a shifting of the equilibrium to the right, thus favouring the extraction of the metals.

Comparison of iron(III) and chromium(III) extraction indicated that iron(III) extraction was favoured over the extraction of chromium(III); however, a near complete extraction (99%) of both metals from the feed solution was achieved. This result was important because using the adequate reaction time and pH value, it was possible to separate both Fe(III) and Cr(III) from Ni(II) (see below), leaving this last element as the only one present in the raffinate after the extraction or transport process had finished. This behaviour was reflected in the values of the overall permeation coefficients, shown in Table 4, as the correspondents to iron(III) were always greater than these of chromium at the same pH values. Moreover, under low proton concentration, diffusion of metal–extractant complexes across the membrane fibres containing DP8R in Exxsol D100 becomes the rate-governing step [28].

With respect to nickel(II) extraction, Figure 6 (bottom centre) indicates that this element is only appreciably extracted (near 48%) at pH 3 and after five hours of reaction, as the percentage of extraction is less than 5% for values of pH below 2. The corresponding overall permeation coefficient values were 0.51 × 10^−5^ cm/s at pH 2.5 and 1.1 × 10^−5^ cm/s at pH 3.

The results derived from Table 4 with respect to the overall permeation coefficient values were consistent with the theory that, using these cation-exchange carriers, metal (solute) transport was inversely related to the proton concentration in the feed phase.

### 3.5. Influence of DP8R Concentration in the Organic Phase on Metal Extraction

As it was easy to understand, an organic phase containing no extractant resulted in negligible extraction or transport of a given solute; thus, the presence of the extractant was a key component of the system to reach success in the extraction or transport process.

**Figure 7:**
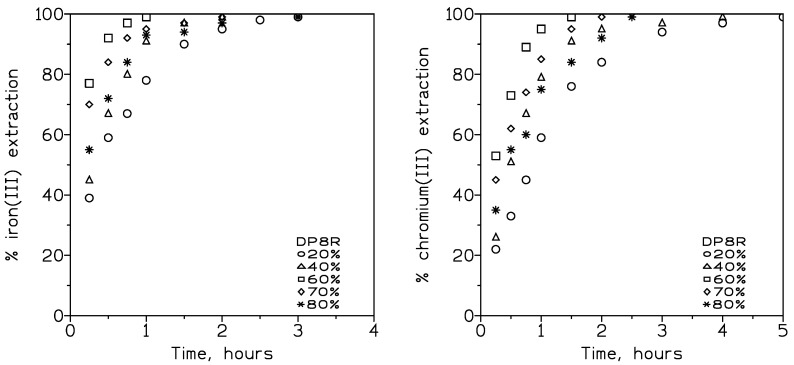
Iron(III) (**left**) and chromium(III) (**right**) extractions at various DP8R concentrations in the organic phase. Temperature: 20 °C.

In this study, the pseudo-emulsion phase consisted of various concentrations (20–80% *v*/*v*) of DP8R in Exxsol D100 and 1 M of sulphuric acid, and this phase was used to extract iron(III), chromium(III), and nickel(II) from a feed phase of pH 2.5. Feed and pseudo-emulsion phases were maintained at 80 cm^3^/min and 180 cm^3^/min, respectively. The results of the investigation are shown in Figure 7, which represents the percentage of metal extraction versus time at the various extractant concentrations used.

These results showed that for both metals, an increase in the extractant concentration from 20 to 60% *v*/*v* was accompanied by an increase in the metal extraction rate at any elapsed time. It should be noted that the reaction time required to reach 99% extraction increased with the decrease in the extractant concentration. In the case of 60% *v*/*v* solution and iron(III), the time required was one hour against three hours for a solution of 20% *v*/*v*, whereas in the case of chromium(III), the time required was of one and a half hours and five hours for extractant concentrations of 60% *v*/*v* and 20% *v*/*v*, respectively, and similar dependences were reported in the literature [29,30,31]. In the case of nickel(II), the same effect was observed, though the percentage of nickel extraction at a fixed time was much lower than the values obtained for both iron(III) and chromium(III) (Table 5).

Table 6 shows the various overall permeation coefficients derived from the extraction results.

As shown in Table 6, in the 20–60% *v*/*v* carrier concentrations range, there was an increase in the metal transport with the increase in the carrier concentration in the organic phase; thus, in this range of concentrations, membrane diffusion becomes dominant. However, at 60% *v*/*v* DP8R concentration, a maximum transport was reached and, consequently, metal transport was dominated by diffusion across the boundary film of the feed phase. At this maximum transport, a limiting overall permeation coefficient can be estimated using the next relationship:

(7) $$P_{\lim}=\frac{D_{f}}{d_{f}}$$

where D_f_ represents the diffusion coefficient of the metal species in the feed phase (averaging value of 10^−5^ cm^2^/s [32]) and d_f_ is the minimum thickness of the feed phase boundary layer. Considering the permeation values showed in Table 7, the values of d_f_ were 2.7 × 10^−2^ cm and 4.2 × 10^−2^ cm for iron(III) and chromium(III), respectively.

As can be seen from Figure 7 and Table 7, at extractant concentrations exceeding 60% *v*/*v*, iron(III) and chromium(III) extraction decreased; thus, metal transport across the liquid membrane did, as well. These results were attributable to an increase in the viscosity of the organic phase resulting from the increase in the extractant concentration in this organic phase [33,34,35].

Assuming that the extractant concentration ([HR]_TOT_) in the membrane fibres was constant, the next equation determined the apparent diffusion coefficients of metal species in the organic phase [36]:

(8) $$D_{\mathrm{org}}^{ap}=\frac{J\cdot d_{m}}{{\left[HR\right]}_{\mathrm{TOT}}}$$

where d_m_ is the fibre wall thickness (3 × 10^−3^ cm) and [HR]_TOT_ 1.8 M (60% *v*/*v*). The metal flux (J) was calculated, under the appropriate experimental conditions, as:

(9) $$J=P_{M}\cdot {\left[M\right]}_{f,0}$$

with P_M_ being the overall permeation coefficient of the metal (Table 6). The values of D_org_^app^ for iron(III) and chromium(III) were 1.1 × 10^−9^ cm^2^/s and 7.8 × 10^−10^ cm^2^/s, respectively.

### 3.6. Influence of the Initial Iron(III) Concentration in the Feed Phase on Metal Transport

In the stainless steel rinse operation, iron(III) has ordinarily been the element that presents the greatest concentration in the water; thus, we also investigated the effect of varying the iron(III) concentration on the transport of the element and also its influence on the transport of the other two elements that accompanied iron(III) in the rinse water.

For this investigation, to the solution containing 0.1 g/L each of chromium(III) and nickel(II), varying (0.1–1 g/L) iron(III) concentrations were added, which were the pseudo-emulsion phase formed by 60% *v*/*v* DP8R in Exxsol D100 and 1 M of sulphuric acid as organic and strippant solutions, respectively.

**Figure 8:**
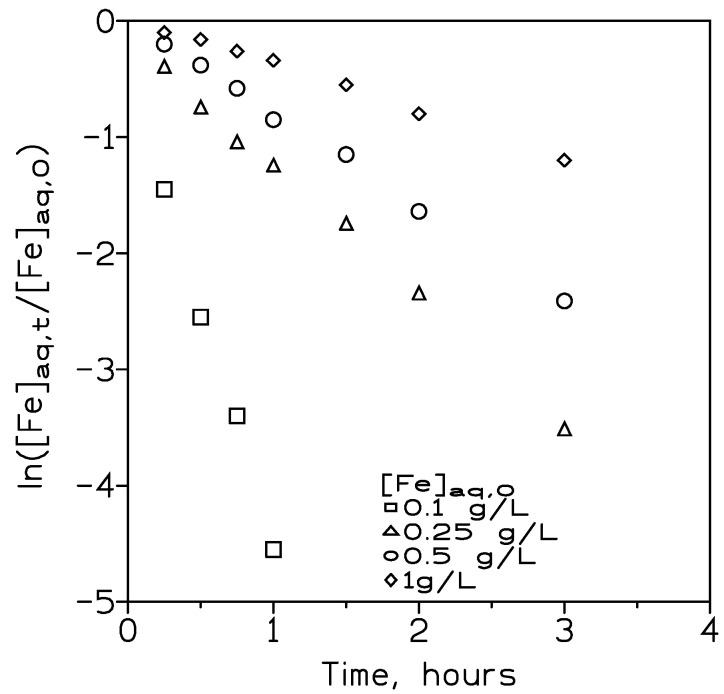
Variation of iron(III) versus time at different initial iron(III) concentrations. Feed phase: pH 2.5. Feed phase flow rate: 80 cm^3^/min. Pseudo-emulsion phase flow rate: 180 cm^3^/min. Temperature: 20 °C.

The results shown in Figure 8 indicate that an increase in the initial iron(III) concentration in the feed phase produced a decrease in iron(III) transport. This negative influence can be related to the fact that with the increase in this concentration in the solution, the organic phase filling the membrane pores of the fibres was saturated with the iron(III)–extractant complex formed in this phase. After, the metal–extractant complex slowly began to diffuse into the bulk of the organic phase, decreasing the mass transfer in the DP8R-Exxsol D100 phase. The increase in the initial iron(III) concentration in the feed phase produced two effects: (i) in the case of iron(III), this increase in the initial concentration resulted in a decrease in iron permeability from 3 × 10^−4^ cm/s to 9.5 × 10^−5^ cm/s for iron(III) concentrations of 0.1 g/L and 1 g/L, respectively, and (ii) in the case of chromium(III) and nickel(II), the permeation (extraction) across the liquid membrane was greatly reduced; this reduction in the permeability can be attributed to two effects: (i) a decrease, due to the increase in iron(III) concentration, in the number of extractant molecules available to be complexed with chromium(III) and nickel(III), and (ii) the aforementioned population or crowding effect due to the presence in the solution of greater iron(III) concentrations [23].

In practical terms, the above situation can be avoided by increasing the membrane surface, and also with longer reaction times [37].

Using Equation (9), the flux values for the different initial iron(III) concentrations investigated in this work can be calculated; Table 7 shows these flux values.

These values indicated that, in accordance with Equation (9), the flux value increased with the corresponding increase in the initial metal concentration in the solution [38], and the transport process was controlled by diffusion in the feed phase. This tendency was maintained up to 0.5 g/L concentration of iron(III), beyond which the metal flux became constant. This was probably due to a saturation of the fibre pores with the iron–extractant complex, which resulted in a lower effective membrane area [39].

### 3.7. Recovery of Iron(III) from the Iron-Loaded Organic Phase

As it was previously described, sulphuric acid was not an effective strippant for this element when loaded in this DP8R extractant. Thus, in this work, once the organic and strip phases disengaged, the iron(III)–organic phase contained in the pseudo-emulsion vessel was separated from the strip solution containing chromium(III), and it was then stripped with 1 M of hydrochloric acid for ten minutes at 20 °C in an O/A ratio of 1. After phase disengagement, the strip solution contained nearly 95% of the iron(III) concentration (0.99 g/L) of the initial organic phase; the concentration factor (see Section 3.2.) for iron(III) was about nine times.

After iron(III) stripping, the extractant can be regenerated to a new non-dispersive extraction step. The reaction responsible for iron(III) stripping can be generalized as:

(10) $$FeR_{3_{org}}+4HCl_{aq}\Leftrightarrow 3HR_{\mathrm{org}}+FeCl_{4_{aq}}^{-}+H_{aq}^{+}$$

The formation of the anionic iron(III)–chloride complex allowed for the stripping of this element. It is worth mentioning here that the non-existence of these anionic complexes in sulphuric (and nitric) acid may be the explanation for why these two mineral acids were not good strippants for iron(III) when acidic extractants were used to extract this metal.

### 3.8. A Proposal for the Treatment of Rinse Waters Using this Membrane Technology

After investigating the feasibility of the use of non-dispersive extraction and strip dispersion with DP8R in Exxsol D100 as a carrier phase for the treatment of rinse waters, several proposals can be drawn:

(i) In all cases, the rinse water must be treated as described in the literature [16] to remove the acid from it.

Proposal 1:

(ii) Separate iron(III) and chromium(III) from nickel(II) in the extraction step; this led to a raffinate containing just nickel(II) and an organic phase containing iron(III) and chromium(III);
(iii) Separation of chromium(III) from iron(III) through selective stripping with sulphuric acid;
(iv) Iron(III) stripping with hydrochloric acid.

Proposal 2:

(ii) Selective extraction of iron(III) and chromium(III) from iron(III), as described in Proposal 1;
(iii) Co-stripping of both elements with hydrochloric acid.

Proposal 3:

(ii) Co-extraction of the three elements; this led to a raffinate that can be recycled to the rinse process as water;
(iii) Co-stripping of the three elements with hydrochloric acid.

There were more sub-variants, but the above seemed to be the simplest. In any case, the selection of one of them must be performed if the recovery of the metals seemed to be attractive from a profitable point of view, and congruent with the necessities of a given plant, etc.

With any of the above proposals, the organic phase of DP8R in Exxsol D100 can be recycled to a new non-dispersive extraction and strip dispersion step.

### 3.9. Estimation of the Mass Transfer Coefficient Values for Metal Transport

Following the same theoretical development as described in the literature [10], a final expression for the overall permeation coefficient can be written as:

(11) $$\frac{1}{P}=\frac{1}{k_{f}}+\frac{r_{i}}{r_{\ln}}\frac{1}{D_{M,f}\cdot k_{m}}$$

where k_f_ is the feed phase mass transfer coefficient, r_i_ is the inner hollow fibre radius, r_ln_ is the hollow fibre log mean radius, D_M,f_ is the metal species distribution ratio, and k_m_ is the membrane mass transfer coefficient. From the above, the expression R = R_f_ + R_m_ can be derived, which expresses that the overall resistance (R) was the sum of the local resistances due to the feed phase diffusion (R_m_) and the membrane diffusion (R_m_).

It was established [40] that the flow velocity of the feed phase governed the individual feed mass transfer coefficient (k_f_):

(12) kf=1.5Dadidi2·vfDa·L13

where D_a_ is the diffusion coefficient of metal species in the feed phase, d_i_ (24 × 10^−3^ cm) is the inner fibre diameter, v_a_ (0.3 cm/s) is the mass flow velocity of the feed phase, and L (15 cm) is the fibre length. At the optimum feed flow of 80 cm^3^/min, the value of k_f_ was established as 6.4 × 10^−4^ cm/s. This coefficient depended on the hydrodynamic conditions, the characteristics of the hollow fibres, and the diffusion of the solute in the feed phase.

This k_f_ value was greater than the overall permeation coefficient values showed in Table 6; thus, the contribution of the fractional resistance due to the feed phase solution (R_f_^0^) to the overall resistance (R) can be calculated as:

(13) $$R_{f}^{0}=\frac{R_{f}}{R}\cdot 100$$

Under the present experimental conditions, the values of R_f_^0^ for the different metals were shown in Table 8.

It can be seen that this step was not the rate controlling of the overall transport process of these metals, except in the case of iron(III) and using an extractant concentration of 60% *v*/*v*, where the contribution of the fractional resistance due to the feed phase was of 59%.

The membrane mass transfer coefficient (k_m_) can be calculated from the next expression [41]:

(14) $$k_{m}=\frac{D_{m}\cdot \varepsilon \cdot d_{lm}}{\tau \cdot d_{m}\cdot d_{o}}$$

where D_m_ (averaging 10^−6^ cm^2^/s) is the diffusion coefficient in the organic phase [42,43,44], ε (0.3) is the membrane porosity, τ (3) is the membrane tortuosity, d_m_ (3 × 10^−2^ cm) is the membrane thickness, and d_lm_ is the log mean diameter of the hollow fibre. Then, the calculated value of k_m_ is 3.0 × 10^−5^ cm/s. This coefficient did not depend on the hydrodynamic conditions applied on a given permeation system; it was only dependent on the fibre characteristics and the diffusion coefficient of the solute–carrier complex formed in the organic solution filling the fibre pores.

The effective diffusion coefficient of the metals–DP8R complexes across the membrane phase can be calculated as [43,45]:

(15) $$D_{eff}=k_{m}\cdot d_{m}\cdot \tau$$

The value of this coefficient was 2.7 × 10^−6^ cm^2^/s, and this coefficient is also dependent on the characteristics of the hollow fibres.

## 4. Conclusions

Non-dispersive extraction and strip dispersion investigations, with a single hollow fibre module for simultaneous extraction and stripping in counter-current mode, were used for the treatment of rinse waters from stainless steel pickling. The results indicated that metal extraction to the organic phase increased with the increase in the extractant concentration in the organic phase, and, consequently, metal transport was governed by membrane diffusion. However, from a certain limiting extractant concentration (60% *v*/*v*), the extraction and, thus, the mass transfer control were shifted to the feed phase. At the highest extractant concentrations, the decrease in the metal extraction (transport) was attributable to an increase in the viscosity of the organic phase. Also, metal extraction was maximized using flow rates of 80 cm^3^/min and 180 cm^3^/min for the feed and pseudo-emulsion phases, respectively. Metal extraction was also pH dependent, and the extraction increased as the pH of the feed solution increased from 1 to 3. This demonstrated that the driving force of metal transport was the difference in proton concentrations between the feed (low) and the stripping (high) phases. Experimental data were used for estimation of various mass transfer coefficients related to the transport system. The stability of this hollow fibre membrane operation was found to be good under controlled flow rates in the feed phase. This technique is a promising alternative to other separation technologies, especially when the metal concentration in the feed solution is low.

## Figures and Tables

**Table 1:** Overall permeation coefficients (single metal feed phase) and rate of recovery in the strip solution.

Metal	P·10^4^, cm/s	%R ^a^
iron(III)	1.2	3
chromium(III)	0.66	95
nickel(II)	0.17	95

^a^ After 5 h.

**Table 2:** Overall permeation coefficients (three metals together in the feed phase) and rate of recovery in the strip solution.

Metal	P·10^5^, cm/s	%R ^a^
iron(III)	6.9	2
chromium(III)	5.1	90
nickel(II)	0.66	90

^a^ After 5 h.

**Table 3:** Overall permeation coefficients and recovery rates in the stripping solution.

Flow Rate, cm^3^/min	P_Fe_·10^4^, cm/s	P_Cr_·10^5^, cm/s	%R_Fe_ ^a^	%R_Cr_ ^a^
17	0.13	1.5	2	90
34	0.69	5.1	3	90
80	1.1	7.1	3	89
100	0.89	6.3	2	91
120	0.40	4.0	3	90

^a^ After 5 h.

**Table 4:** Overall permeation coefficients at various pH values of the feed phase and recoveries in the stripping solution.

pH	P_Fe_·10^−4^, cm/s	P_Cr_·10^−5^, cm/s	%R_Fe_ ^a^	%R_Cr_ ^a^
1	0.40	0.40	2	90
2	0.66	0.46	2	93
2.5	1.1	0.71	3	89
3	2.5	1.2	3	92

^a^ After 5 h.

**Table 5:** Percentages of extraction for Fe(III), Cr(III), and Ni(II).

Time, h	Fe(III)	Cr(III)	Ni(II)
0.5	92	73	10
1	99	95	13
3	99	99	24
5	99	99	36

Organic phase: 60% *v*/*v* DP8R in Exxsol D100. Other experimental variables as in Figure 7.

**Table 6:** Overall permeation coefficients at the various carrier concentrations investigated.

Extractant, % *v*/*v*	P_Fe_·10^−4^, cm/s	P_Cr_·10^−4^, cm/s	P_Ni_·10^−6^, cm/s
20	1.1	0.71	4.9
40	1.8	1.2	5.7
60	3.7	2.4	7.4
70	2.0	1.7	no data
80	1.2	0.97	no data

**Table 7:** Iron(III) flux values at various initial metal concentrations in the feed phase.

[Fe]_f,0_, g/L	P·10^−4^, cm/s	J·10^−9^, mol/cm^2^·s
0.1	3.7	0.67
0.25	2.8	1.3
0.5	1.9	1.7
1	0.95	1.7

**Table 8:** Values (%) of the contribution of the fractional resistance due to the feed phase.

Extractant, % *v*/*v*	Fe(III)	Cr(III)	Ni(II)
20	17	11	0.8
40	29	19	0.9
60	59	38	1

## Data Availability

Data is contained in this article.
